# Hybrid Cuckoo Search–Bees Algorithm with Memristive Chaotic Initialization for Cryptographically Strong S-Box Generation

**DOI:** 10.3390/biomimetics10090610

**Published:** 2025-09-10

**Authors:** Sinem Akyol

**Affiliations:** Software Engineering Department, Engineering Faculty, Firat University, 23319 Elazig, Turkey; sakyol@firat.edu.tr

**Keywords:** Bees Algorithm, Cuckoo Search Algorithm, chaotic maps, Memristive chaotic map, Substitution Boxes (s-boxes), nonlinearity

## Abstract

One of the essential parts of contemporary cryptographic systems is s-boxes (Substitution Boxes), which give encryption algorithms more complexity and resilience due to their nonlinear structure. In this study, we propose CSBA (Cuckoo Search–Bees Algorithm), a hybrid evolutionary method that combines the strengths of Cuckoo Search and Bees algorithms, to generate s-box structures with strong cryptographic properties. The initial population is generated with a high-diversity four-dimensional Memristive Lu chaotic map, taking advantage of the random yet deterministic nature of chaotic systems. This proposed method was designed with inspiration from biological systems. It was developed based on the foraging strategies of bees and the reproductive strategies of cuckoos. This nature-inspired structure enables an efficient scanning of the solution space. The resultant s-boxes’ fitness was assessed using the nonlinearity value. These s-boxes were then optimized using the hybrid CSBA algorithm suggested in this paper as well as the Bees algorithm. The performance of the proposed approaches was measured using SAC, nonlinearity, BIC-SAC, BIC-NL, maximum difference distribution, and linear uniformity (LU) metrics. Compared to other studies in the literature that used metaheuristic algorithms to generate s-boxes, the proposed approach demonstrates good performance. In particular, the average value of 109.75 obtained for the nonlinearity metric demonstrates high success. Therefore, this study demonstrates that robust and reliable s-boxes can be generated for symmetric encryption algorithms using the developed metaheuristic algorithms.

## 1. Introduction

The technique of transforming legible plaintext into ciphertext—a form that is unintelligible or meaningless—is known as cryptography. The science of encryption, or cryptography, is essential to maintaining information security in the current digital era. Modern computer systems use data encryption algorithms with symmetric keys to ensure integrity, authentication, confidentiality, and other information security services [1,2,3]. Both the sender and the recipient encrypt and decrypt data using the same secret key in symmetric algorithms. S-boxes are the only nonlinear key component in the symmetric encryption technique AES. They also make the connection between the ciphertext and the key as complicated as possible and obscure it, which makes them resistant to cyberattacks [4].

Because s-boxes increase complexity due to their nonlinear structure, they are used in encryption algorithms to increase security. S-boxes are particularly used in symmetric encryption algorithms because they increase resistance to linear attacks by adding a nonlinear structure. This robust structure makes it difficult for attackers to figure out how the key and the ciphertext are related during the encryption process. Another reason is that s-boxes provide variety and complexity. They make encryption methods more unpredictable by adding a complex link between input and output. Furthermore, by offering defense against attacks like differential and linear cryptanalysis, s-boxes’ sturdy architecture raises the fundamental degree of security.

A reliable s-box design requires striking a compromise between mathematical features and practical efficiency. A well-designed s-box allows for big changes in the output from small changes in the input values. Chaotic systems and metaheuristic optimization algorithms have been used in recent years to develop s-box creation techniques [5]. S-boxes are dynamically produced using mathematical models in the Linear Trigonometric Transformation approach [6]. Latin squares and chaotic arrays are used to build high-security s-boxes in architectures based on Exact Latin Squares [7].

Galois fields are used to build s-boxes with bijective features in Mobius Transformation and Permutation Functions [8]. In Oblique Function and Chaos Systems, structures with high nonlinearity are designed using chaotic maps and oblique functions [9]. Optimization methods like the Artificial Bee Colony Algorithm, Cuckoo Search Algorithm (CSA), and chaotic maps are merged in Discrete Chaotic Maps and Metaheuristic Methods [10,11]. Nonlinear s-boxes are developed with irreducible polynomials and multiple transformations in Affine Transformations [12]. Hash functions are used by FPGA circuitry to convert the physical randomness information from the R-S latch’s metastable state circuits into s-boxes [13]. Targeting linear components, the chaotic data that is produced by combining DNA-based coding approaches with the 2D hyper-chaotic Schaffer map is processed and transformed into s-boxes [14].

The creation of s-boxes frequently uses evolutionary algorithms. Their ability to identify solutions near the global optimum despite the solution space’s width makes them a particularly useful tool in s-box design. These algorithms try to obtain optimal structures by using the cryptographic criteria of s-boxes as a fitness function. By avoiding local optima in the solution space, more complicated and safe s-boxes can be obtained since evolutionary methods are unpredictable and adaptable. Furthermore, the algorithms’ search skills are further enhanced and better results can be produced than with the traditional approaches suggested in the literature because of their hybrid versions. For evolutionary algorithms to effectively scan the solution space, the initial population’s diversity is crucial. Chaotic maps are commonly utilized in the initial population production to guarantee the initial population’s diversity and to provide a more balanced dispersion in the solution space [15]. Compared to other maps, Liu and his colleagues claimed that the Memristive chaotic map is more complex, has a larger attractor space, and a wider chaotic range [16].

This study used the complementing advantages of the Bees Algorithm (BA) and the CSA to create a hybrid evolutionary technique. As an initial population, high-diversity s-boxes were created using the four-dimensional Memristive Lu chaotic map. The evolutionary algorithms used in this study are based on nature-based approaches inspired by biological systems. The BA was developed based on the behavior of honeybees in nature, exploring food sources and sharing their locations with other bees in their colonies. Similarly, the CSA was developed based on the cuckoos’ reproductive strategy of laying their own eggs in other birds’ nests. These algorithms, inspired by such biological systems, enable more efficient scanning of the solution space and prevent premature convergence. Furthermore, the use of chaotic maps in generating the initial population enhances the randomness and diversity in nature, enabling the generation of more balanced and diverse solutions. In this respect, this study presents an interdisciplinary approach that aligns with biomimetic design principles in cryptographic s-box generation.

To produce cryptographically robust s-boxes, hybrid CSBA was used to optimize these created s-boxes. The suggested approach is directed by a fitness function that targets linear components and is intended to provide security criteria like balanced distribution and high nonlinearity.

The main contributions of this study are as follows:

- In this study, it is aimed to overcome the limitations encountered in the s-box design by combining the strengths of both chaotic systems and metaheuristic algorithms.
- A new method, CSBA, formed by hybridizing the BA and the CSA, is proposed.
- With this hybrid approach, it is aimed to both increase cryptographic security and optimize computational efficiency.
- First, s-boxes were created using four-dimensional Memristive Chaotic Maps. These s-boxes were given as the initial population to the heuristic algorithms.
- As a fitness function, nonlinearity values were calculated using the Walsh-Hadamard spectrum and these values were evaluated to optimize the performance of the algorithm.

The article’s progression is as follows. In the study’s Section 2, literature studies on the production of s-boxes are included. In Section 3, the Heuristic Algorithms used and the modifications made to them are mentioned. In Section 4, the results obtained in this study are given and interpreted. In Section 5, the study is concluded and future studies are mentioned.

## 2. Related Works

Shadab et al. proposed the Hawkboost algorithm, which combines Harris Hawks optimization and Booster algorithm, to optimize s-box generation by efficiently navigating the search space and increasing nonlinearity via local operators. They addressed the challenges in s-box design with low computational effort. The suggested Hawkboost algorithm performs better in s-box generation, and the suggested s-box successfully satisfies the cryptographic property requirements. However, generating highly nonlinear s-boxes is NP-hard and the large permutation search space complicates s-box optimization [17].

The s-box generation process proposed by Baowidan et al. improves nonlinearity and cryptographic parameters using heuristic group-based optimization. This method includes group actions in Galois fields, resulting in a more robust s-box with a nonlinearity score of 112 compared to traditional s-boxes. The results show high nonlinearity score and strong cryptographic robustness. This method is effective against statistical and cryptanalytic attacks and is resistant to various cryptographic attacks. It also contributes to the reduction in statistical vulnerabilities that may arise in encryption [18].

By fusing the exploratory potential of population-based approaches with the local search efficiency of the hill climbing (HC) algorithm, Kuznetsov et al. developed the Hybrid Population-Based HC (HPHC) method. They generated s-boxes using this suggested approach and received a score of 104 on the Nonlinearity criterion [19].

In another work, Kuznetsov et al. optimized the HC method in the s-box creation process, reducing the iteration number required to construct s-boxes with a bijective and nonlinearity value of 104 from 65,000 to approximately 50,000. This optimization significantly increases the processing efficiency and strengthens cryptographic strength. However, it has difficulties such as high computational cost for random s-box generation and the complexity of obtaining s-boxes with the desired cryptographic properties. The results obtained: Although a nonlinearity value of 104 was targeted, s-boxes with a nonlinearity score of 102 were obtained faster. The processing complexity in s-box generation was reduced by optimizing the HC algorithm [20].

In a different work, Kuznetsov et al. modified the Simulated Annealing (SA) technique to generate s-boxes with strong nonlinearity in 8-bit size, and they presented a new fitness function. The proposed new cost function was able to target the coefficients causing the nonlinearity by focusing mostly on values greater than 40 in the Walsh–Hadamard spectrum. The studies yielded s-boxes with a 100% success rate and a nonlinearity value of 104 after an average of 83,000 iterations [21].

Namuq optimizes the s-box generation process using 3D chaotic maps and increases security with high nonlinearity, optimal avalanche effect and strict avalanche criterion. In this way, lower linear and differential probability values are obtained compared to traditional methods and resistance to cryptographic attacks is increased. As a result, significant improvements in nonlinearity and differential uniformity are achieved in this study. Lower linear probability and differential probability values are obtained [22].

Yang et al. created the initial population by creating chaotic sequences with high Lyapunov exponent and complex dynamics using a new four-dimensional hyperchaotic system developed based on the Lorenz system. A Particle Swarm Optimization (PSO) technique built on the ideas of SA was used to optimize this initial s-box after it had been combined with the Arnold transformation. When the results are examined, the maximum value of 110 was obtained in the Nonlinearity criterion, while the average value was 107 [23].

Jassim designed 8 × 8 s-boxes with the African Buffalo Optimization technique and a random function. Evaluating the results of the study: When looking at the Nonlinearity criterion, it is seen that the value of 107 was obtained [24].

Wang et al. produced s-boxes with high security performance using digital cascade chaotic mapping and the developed Memorable SA method. In their study, it was shown that s-boxes optimized using a fitness function based on nonlinearity, difference uniformity and SAC/BIC criteria were both cryptographically strong and efficient in FPGA-based applications. When the results were examined, it was observed that the average score in the nonlinearity criterion was 108 [25].

To increase the nonlinearity value of s-boxes, Freyre-Echevarría et al. created a fitness (cost) function that is independent from outside parameters. In experiments conducted with genetic-tree, local search and HC algorithms, the highest nonlinearity value was obtained as 104 over randomly initialized 8×8-dimensional s-boxes. The proposed function aims to ensure that the coefficients on the Walsh–Hadamard spectrum do not exceed the SCV limit, allowing similar results to be achieved with fewer solution evaluations compared to previous studies [26].

Ustun and his colleagues created a new s-box employing the mutation and crossover operators of a genetic algorithm expressed in real numbers and integrated it into the picture encryption process. The developed s-box has neither fixed nor inverse fixed points, a short iteration loop, and strong cryptographic properties (average nonlinearity value of 111.25). In the encryption algorithm, permutation, diffusion, and bit inversion operations were performed using this s-box along with a two-dimensional hyperchaotic Styblinski–Tang map. Security analyses (NPCR, UACI, entropy, histogram, correlation, key sensitivity, etc.) have shown that the approach is very resistant to various sorts of assaults [27].

Ustun and Sahinkaya proposed a new approach to secure s-box design that combines the 2D-Zettle chaotic map with the Artificial Bee Colony (ABC) algorithm. S-boxes were optimized separately for RGB channels using a chaotic map and the ABC method. The binary multi-neighborhood ABC technique was used to generate encryption and decryption keys. The results showed a nonlinearity criterion value of 110.5 [28].

Lawah et al. used the Crossover Grey Wolf Optimizer method, which is based on the grey wolf optimization algorithm, to create 8 × 8 s-boxes with strong cryptographic features. A range of cryptographic performance indicators, such as bit independence, bijective property, linear probability, strict avalanche, and I/O XOR distribution, were used to evaluate the proposed method. When the results were analyzed, they found an average value of 109 in the Nonlinearity criterion using the Crossover Grey Wolf Optimizer method [29].

Alhadawi et al. used the CSA and discrete chaotic maps to create a novel 8 × 8 s-box generating method. Significant initial diversity was produced by using discrete chaotic maps, which also lessened the CSA’s propensity to become trapped in local minima. When the results were examined, it was shown that the obtained s-boxes exhibited high cryptographic performance with a nonlinearity value of 108.5 and superior results were obtained when compared to existing chaotic/optimization-based methods [10].

Zamli et al. created s-boxes using a new metaheuristic algorithm, SCMTTA (Selective Chaotic Maps Tiki-Taka Algorithm), in which five different chaotic maps (logistic, tent, chebyshev, sine, and singer map) were adaptively combined. In the proposed method, the selection between chaotic maps was performed with a success-based reward–punishment mechanism. The SCMTTA algorithm, thanks to the variety of chaotic maps, avoids local minima and achieves high nonlinearity values, and an average of 109.25 is obtained in the nonlinearity value [30].

Using an enhanced PSO algorithm, Hematpour and Ahadpour have used chaotic s-box creation. Several ergodic chaotic maps were used in the study to improve the initial population and PSO updates. The s-boxes produced with the enhanced PSO were optimized using two distinct fitness functions (the mono-bit test *p*-value and nonlinearity). The findings showed that the s-boxes produced using the suggested method had a maximum nonlinearity value of 108 [31].

Chaos-based methods are highly sensitive to initial conditions and exhibit complex dynamic behavior. Therefore, these methods have become an important research area in s-box generation in recent years. There are many studies in the literature where chaotic maps are used in encryption algorithms. Yang et al. developed a global amplitude controlled discrete hyperchaotic memristive map based on the hyperbolic tangent function for image encryption [32]. Boobalan and Gurunathan Arthanari designed a reversible s-box using a Lorenz and Chua-based hybrid chaotic key generator to increase security in image encryption. The obtained s-box showed strong complexity and propagation properties [33]. Ustun and Sahinkaya used the artificial bee colony algorithm and 2D Zettle chaotic maps to generate cryptographically strong s-boxes [28]. Qazi et al. used a unique 1D chaotic map defined by a trigonometric function chain to generate a chaotic-based dynamic s-box [34].

Deng et al. developed a hyperchaotic memristive Hopfield neural network architecture that can generate complex multi-wing hidden hyperchaotic attractors by integrating a dual memristive component into the traditional Hopfield neural network structure for the security of medical images [35]. Yao et al. designed an asymmetric memristive hopfield neural network by developing a new memristor model with high stability and high tunability. When they examined the chaotic dynamics of the proposed system using basic dynamic analysis methods such as equilibrium point stability, bifurcation diagrams, and Lyapunov exponents, they found that it has complex properties [36]. Deng et al. developed a new memristive tabu learning neuron model by integrating a memristor with a piecewise nonlinear state function into the tabu learning neuron model. This model can generate a double-wing chaotic butterfly structure, and the number of wings can be easily changed by adjusting the state function of the memristor [37].

## 3. Theoretical Background

### 3.1. Four-Dimensional Memristive Hyperchaotic Map

The Memristive Lu chaotic map is an extended version of the classical Lu system with Memristive effects, which outperforms traditional chaotic systems. The four-dimensional Memristive Lu model exhibits chaotic dynamics under certain parameters and is based on four state variables that are updated at each iteration [38]. The iterative form of the map is given in Equations (1) and (2).

(1) $$\begin{array}{c} x_{t+1}=x_{t}+a\left(y_{t}-x_{t}\right)\times ∆t\\ y_{t+1}=y_{t}+(cx_{t}-x_{t}z_{t}{+dy}_{t})\times ∆t\\ \begin{array}{c} z_{t+1}=z_{t}+(x_{t}y_{t}-bz_{t})\times ∆t\\ w_{t+1}=w_{t}+({-w}_{t}+x_{t}z_{t})\times ∆t \end{array} \end{array}$$

(2) $$z_{m+1}=mod\left(\left|x_{t+1}+y_{t+1}+z_{t+1}+w_{t+1}\right|,1\right)$$

**Figure 1:**
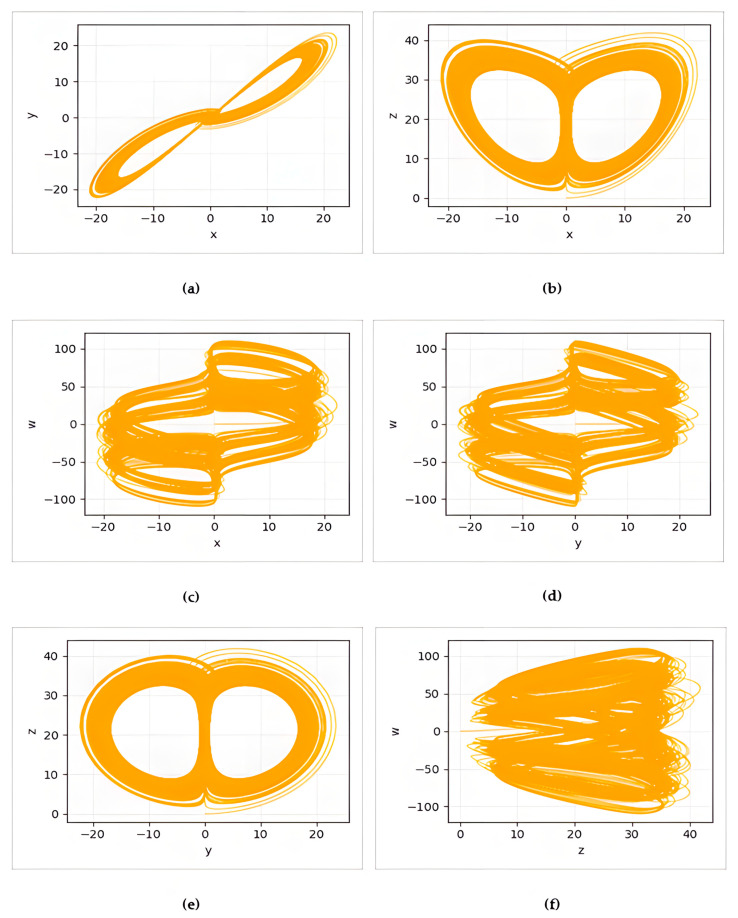
(**a**) x-y, (**b**) x-z, (**c**) x-w, (**d**) y-w, (**e**) y-z and (**f**) z-w phase diagrams of the 4D Memristive Lu chaotic map.

Here, $x_{t}$, $y_{t}$, $z_{t}$ and $w_{t}$ are the time-dependent state variables of the system. In this study, the coefficients for the Memristive Lu chaotic map were set as *a* = 36, *b* = 3, *c* = 20, *d* = 0.5, as reported in [39]. ∆t shows the step size and is taken as 0.01 in this study. The phase diagrams of the 4D Memristive Lu chaotic map are given in Figure 1.

The chaotic condition of the system is confirmed by the complicated and non-periodic trajectories seen in the initial and system parameters’ phase space. The complex, dense, and non-periodic trajectories observed in the phase diagrams in Figure 1 clearly reveal the 4D chaotic system’s dynamic evolution. This dynamic evolution demonstrates the system’s constant unpredictable and non-repetitive tendency toward multidimensional attractors and its significant sensitivity to initial conditions. This complex behavior of the system provides an ideal basis for the unpredictability and high randomness properties sought in cryptographic applications.

In the proposed method, the diversity of the initial population is critical for evolutionary algorithms to explore a large search space without premature convergence and to generate robust s-boxes. Therefore, in our study, the initial population was created with a four-dimensional memristive Lu map, which exhibits more complex dynamics, instead of classical/familiar one-dimensional chaotic maps (e.g., Logistic, Tent) to increase randomness and homogeneity. This system, thanks to its high dimensionality and memristive structure, produces richer dynamic patterns and has the potential to increase the diversity of the initial individuals. These features contribute to the proposed hybrid algorithm (CSBA) exploring the search space in a more balanced manner, and the resulting s-boxes provide competitive results in the nonlinearity, SAC, and BIC criteria.

### 3.2. Bees Algorithm

During the harvest season, bees send some of themselves as scouts to explore suitable flower areas in the surrounding area. In order to maximize the colony’s food collection efficiency, scouts use a unique dance to alert other bees to the location and productivity of a quality region when they return to the hive. The Bee Algorithm was inspired by these food search behaviors of honey bees [40].

The number of regions to be chosen for neighborhood search (m), the number of scout bees (n), the number of areas deemed the best among these m regions (e), the number of bees to be sent to the remaining (m−e) regions (nsp), the number of bees to be directed to the best e region (nep), the initial size of each patch (ngh), and the stopping criterion are some of the fundamental parameters that must be set in order to use the algorithm. The process determines the fitness values of the sites that n scout bees visited after they were randomly assigned to the search space. A neighborhood search is conducted in the vicinity of the m regions with the highest fitness value. By sending more bees (nep) to the optimal e region and fewer bees (nsp) to the other m−e regions, the density of this search is changed. In this way, the algorithm continues to explore new regions and aims to reach a solution by conducting more detailed research in the most promising areas.

**Figure 2:**
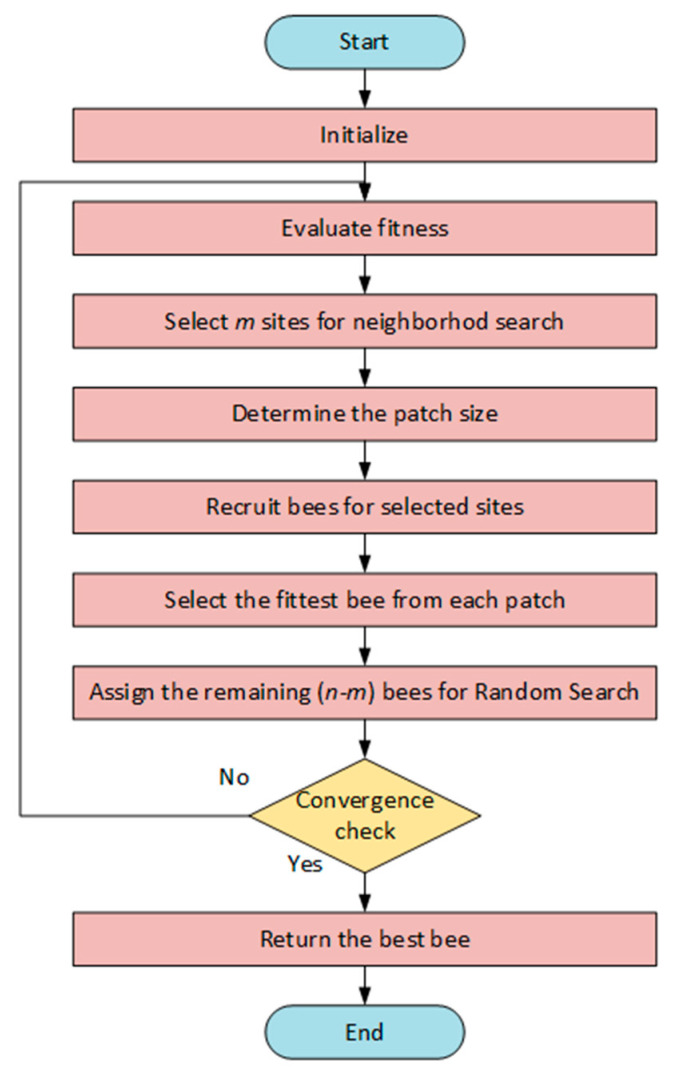
The flow chart of BA.

In the next step, only the bee that reaches the highest fitness value (the best bee) for each patch is transferred to the next generation. Although there is no such restriction in nature, this restriction is applied in order to narrow down the search space. If the searches made in a patch—even if the neighborhood size is changed—do not produce a better result, this region is considered to be a local optimum. In this case, the patch is abandoned and this process is called “abandoning areas that do not produce new information”. The population’s surviving bees are then assigned to find fresh potential solutions after being dispersed at random throughout the search space. The best individuals in the chosen patches and the scout bees that conduct random searches make up the new population at the conclusion of each iteration. Until the halting requirement is satisfied, this cycle keeps going. In the case of a constrained solution problem, the fitness is decreased below a predetermined threshold by applying a fixed penalty to the solutions that break the constraint [40]. In Figure 2 the flow chart of BA is shown.

### 3.3. CSA

Xin-SheYang and Suash Deb developed the CSA, a metaheuristic optimization technique, in 2009 after being influenced by the parasitic reproductive strategies of some cuckoo species. While host birds care for their own young, these species lay their eggs in the nests of other birds. Some species specifically evade discovery by mimicking the color and appearance of their eggs to those of their host species. This behavior of cuckoos is adapted to the solution search and selection process in the algorithm [41].

The following three fundamental guidelines govern how the algorithm operates:

- Every cuckoo lays an egg, meaning that every person creates a fresh answer in the search space, and this solution tries to replace a randomly selected existing solution (nest).
- High-quality solutions (good eggs) are passed on to the next generation.
- A portion of the solutions noticed by the host (at the rate of $P_{a}\in \left[0,1\right]$) are replaced by randomly generated new solutions.

Lévy flights are used to generate new solutions. Lévy flights are a search strategy defined by random steps with a heavy-tailed distribution. This strategy increases the capacity to explore large areas and prevents solutions from getting stuck in local minima. A new solution $x_{i}^{\left(t+1\right)}$ is generated as in Equation (3).

(3) $$x_{i}^{\left(t+1\right)}=x_{i}^{\left(t\right)}+\alpha \cdot Levy(\lambda )$$

Here, $x_{i}^{\left(t\right)}$ is the solution of the ith cuckoo at time t, α>0 is step size (usually taken as 1), and Levy(λ) is a randomly selected step according to the Lévy distribution. The Lévy distribution is a heavy-tailed distribution and is expressed as in Equation (4).

(4) $$Levy\sim u=t^{-\lambda },\;(1<\lambda \leq 3)$$

This distribution allows the algorithm to perform both local and global searches in the solution space. While long steps allow for extensive searches, short steps allow for local examinations [42].

At the end of each iteration, the best solutions are preserved, low-quality solutions are eliminated with probability $P_{a}$ and replaced with randomly generated new individuals (new nests). Thus, the population is constantly renewed and the chance of reaching better solutions increases. If the solution contains a constraint violation, the fitness value is reduced by penalizing as in Equation (5).

(5) $$f_{penalize}=f\left(x\right)+penalty$$

**Figure 3:**
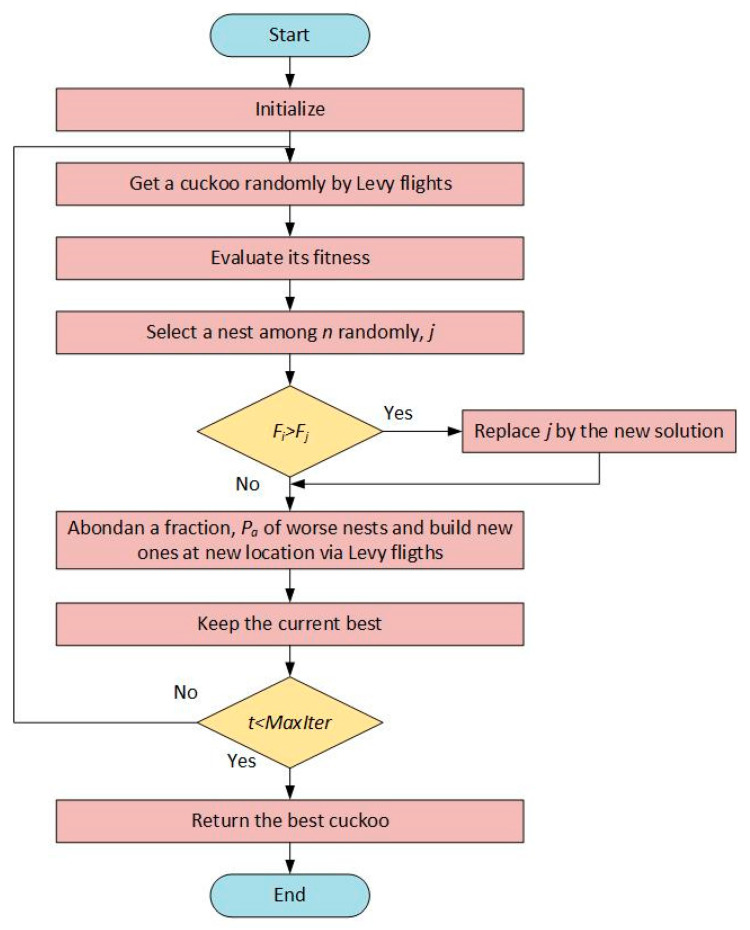
The flow diagram of CSA.

This penalty function ensures that the algorithm works effectively in constrained problems [42]. The flow diagram of the CSA algorithm is shown in Figure 3.

## 4. Proposed Method (A Memristive-Initiated Hybrid Cuckoo Search and Bees Algorithm)

Because of their readily adjustable architecture, intelligent metaheuristic algorithms are widely applied in a variety of complicated real-world issues of diverse types [43]. In this study, hybrid CSBA, which is a hybrid version of Bees Algorithm and Cuckoo Search algorithms, is proposed to produce s-box structures with strong cryptographic properties. The convergence behavior of the hybrid algorithm, as is common in evolutionary algorithms, may vary depending on certain random number sequences due to the stochastic nature of the algorithm. This algorithm also does not provide analytically guaranteed results, and its performance is shaped depending on the randomness sources used and parameter interactions [15]. In this study, the irregular yet deterministic feature of chaotic systems was exploited to generate random numbers using chaotic maps. In this context, it was aimed to obtain a wider spread in the solution space by using a four-dimensional Memristive Chaotic Map in the creation of the initial population.

### 4.1. Initialization of the Population

The initial individuals of the population were generated using chaotic number sequences generated by the four-dimensional Memristive Chaotic Map given in Equations (1) and (2). These sequences were transformed into unique permutations in the range of 0–255 to obtain valid s-box structures. The equation for creating the initial population is given in Equation (6).

(6) $$X_{i,j}=round\left(255\cdot z_{m+1}\right)$$

This method increases the algorithm’s global search capability by initially ensuring a balanced and diverse distribution of the population in the solution space. The pseudocode for the first step is shown in Table 1.

The UsedValues list is used to ensure that the s-boxes consist of unique values in the range of 0–255. Here, the value $z_{m+1}$ generated by Equation (6) is checked to see if it is included in the UsedValues list. If it is not, it is included in the s-box permutation and added to the UsedValues list. If it is, the generation of a new chaotic value continues. This ensures that the generated s-box consists of unique numbers in the range of 0–255.

### 4.2. Hybrid Cuckoo and Bees Search Algorithm

In this study, a hybrid method, the CSBA algorithm, which combines the strengths of BA and CSA, is proposed. Each iteration of the algorithm has a two-stage structure. In the first stage, new solutions are produced using the Levy Flight step in CSA. With this step, it is possible to explore wider areas in the solution space with random but directed steps. In the second stage, a more detailed search is performed in the search area with the local search strategy of BA.

#### 4.2.1. CSA Step

In this step, a new solution is created for each individual based on the Lévy flight. New individuals are calculated using Equation (3). The generated solution is compared with the existing individual and the search process is continued by selecting the better one.

#### 4.2.2. BA Step

The most successful m individuals in the population are determined and a neighborhood search is performed around these individuals. A more intensive search is performed around the best e individual (nep individual) and a less frequent search is performed around the other m−e individuals (nsp individual). The best individual in each region is transferred to the next generation. This structure contributes to both detailed search and preservation of diversity in the solution space.

#### 4.2.3. Abandonment of Local Optima

It is assumed that the areas where individuals cannot be improved as a result of the neighborhood search are local maxima in the solution space. Therefore, in order to avoid local maxima, these individuals are abandoned and new individuals are randomly added in their place. The purpose of this phase is to keep the algorithm from being trapped in the local optimum.

In heuristic algorithms, the balanced operation of the exploration and exploitation phases is crucial. In the proposed hybrid CSBA, balancing the exploration and exploitation phases relies on the interaction of the parameters used in the CSA and BA steps. First, the Levy flight step size (α) used in the CSA step enables exploration in different regions by making long jumps in the solution space. If a large α value is selected, the algorithm searches a broader range of the search space, while a small value searches an area closer to the current solution. In this study, an α value of 1.5 was selected to enable the exploration phase to be performed with the CSA step.

In the BA step, the m, e, nep, and nsp parameters determine the local search intensity. In this study, m = 5, e = 2, nep = 20, and nsp = 10 were selected. These values were chosen based on the parameters presented in a literature review of BA [44]. Increasing the nep and nsp values increases exploitation in good regions while also contributing to the preservation of diversity.

In the initial iterations, the exploration phase was focused, followed by the exploitation phase, which then proceeded to search the neighborhood of the current solution. Additionally, the abandonment of local optima phase was added, generating random solutions, thus reducing the risk of getting stuck in a local optimum. This enabled the algorithm to perform a certain level of exploration even at a high-exploitation stage. With this proposed hybrid algorithm, an extensive exploration step is performed in the early stages to identify potential good regions. In subsequent stages, intensive exploitation of these regions is performed to find the best solution.

**Figure 4:**
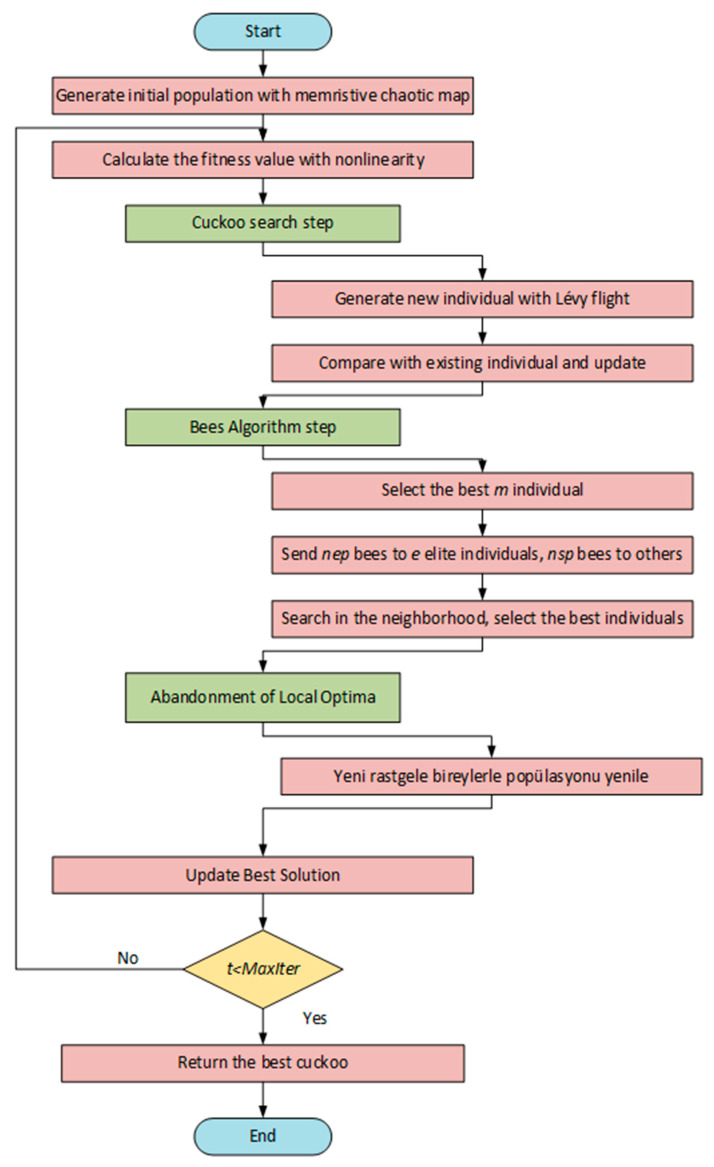
The flow diagram of proposed model.

This two-component structure of the algorithm allows scanning large areas at the beginning and improving around quality solutions in the later stages. Furthermore, each iteration preserves the best answers, guaranteeing algorithm stability and speeding up the convergence process. Figure 4 displays the suggested model’s flow diagram.

### 4.3. Fitness Function

In this paper, the fitness function was the nonlinearity value. The population’s nonlinearity values, or s-boxes, were used to measure each person’s fitness, and the s-box with the highest fitness value was offered as the answer. The calculation of the nonlinearity value is given in Equation (7).

(7) $$N_{f}=2^{n-1}\left(1-2^{n}{max}_{w\epsilon GF\left(2^{n}\right)}|{WS}_{≺f≻}\left(w\right)|\right)$$

Here, the Walsh spectrum value $f\left(x\right)$ is calculated as in Equation (8).

(8) $${WS}_{≺f≻}\left(w\right)=\sum_{x\epsilon GF(2^{n})}{\left(-1\right)}^{f(x)⊕x\cdot w}$$

Here $w\;\epsilon \;GF(2^{n})$. x·w represents the dot product of x and w and is calculated as in Equation (9).

(9) $$x\cdot w=x_{1}⨁w_{1}+x_{2}⨁w_{2}+\cdots +x_{n}⨁w_{n}$$

## 5. Experimental Results

This study presents experimental findings for the s-boxes produced by BA and CSBA, evaluating their cryptographic performance using metrics such as nonlinearity, Strict Avalanche Criterion (SAC), Linear Approximation Probability (LP), Bit Independence Criterion (BIC), and Input/Output XOR Distribution. Both algorithms were executed with a population size of 30 over 100 iterations.

**Figure 5:**
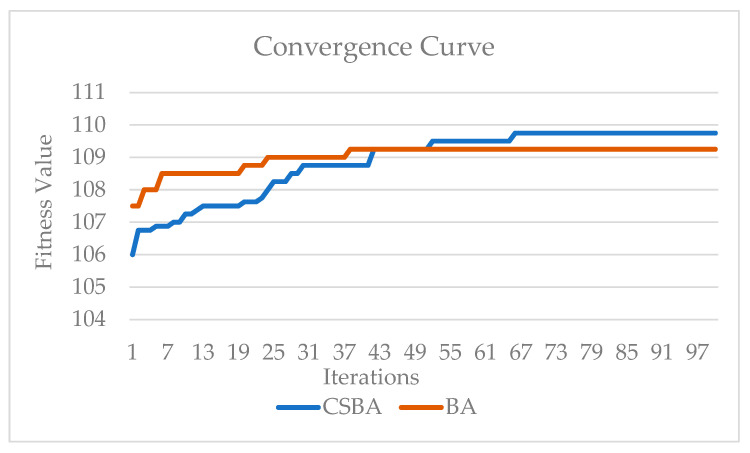
Convergence curve for CSBA and BA.

The convergence behavior of the CSBA and BA algorithms over 100 iterations in comparison to the preceding one is displayed in Figure 5. An examination of the convergence curves reveals that the improved CSBA algorithm reaches the optimal solution faster and exhibits stable convergence. This is due to the successful integration of the powerful global search capabilities of CSA and the local optimization capabilities of BA.

Despite the instability and fluctuations observed in the BA algorithm, particularly as the number of iterations increases, the CSBA algorithm progressed more smoothly toward the optimal solution. This enabled the hybrid approach to perform an extensive search in the early stages and then refine the solutions precisely. Therefore, the convergence curve in Figure 5 demonstrates that the proposed CSBA algorithm offers both a more efficient and effective optimization process.

An s-box generated using BA is presented in Table 2. Each input of the s-box is mapped to a unique (bijective) output in the range 0–255. This demonstrates the successful generation of a cryptographically valid and usable s-box. All 256 different output values in the s-box are used only once, demonstrating that the bijectivity property is satisfied.

Table 3 displays the s-box that was produced by the suggested CSBA hybrid algorithm. Additionally, there are 256 distinct values in this table, all of which fall between 0 and 255. The created s-box satisfies the fundamental cryptographic requirements since the bijectivity property is fully satisfied for this s-box.

A comparative analysis of the nonlinearity metrics of the s-boxes generated by CSBA and BA is provided in Table 4. In this table, N_1_–N_8_ columns represent the nonlinearity scores of eight independently generated s-boxes. Among the key indicators used to evaluate an s-box’s resistance to linear cryptanalysis, nonlinearity plays a central role. The table illustrates that both algorithms successfully produced s-boxes exhibiting strong nonlinearity characteristics.

**Figure 6:**
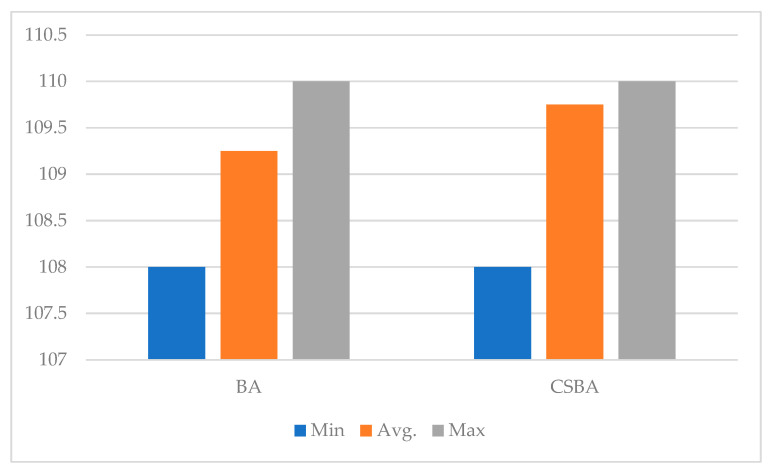
Nonlinearity score measurement for s-boxes produced by CSBA and BA.

The graphical distribution of the nonlinearity values of the s-boxes produced using CSBA and BA is displayed in Figure 6. This figure provides a comparison of the algorithms’ production performance in terms of cryptographic quality.

The nonlinearity values of the s-boxes produced by the BA algorithm range from 108 to 110, with an average value of 109.25, as Table 4 and Figure 6 demonstrate. While these values are quite satisfactory, the relatively low values of some individual s-boxes, such as 108, indicate that BA can occasionally be trapped in local minima. The s-boxes generated with CSBA demonstrate more consistent and higher performance, with seven s-boxes at 110 and one at 108. The average value is 109.75, which is higher than BA. This demonstrates that CSBA produces more optimal solutions by more effectively balancing exploration and exploitation.

Table 5 and Table 6 present the SAC values for BA and CSBA, respectively. SAC is a crucial metric that assesses whether a change in one bit of the input affects roughly half of the output. The values in the tables ought to be near the optimal value of 0.5 for SAC.

Table 5 details the SAC measurements of the s-box generated with BA. The measurement results show that SAC values are very close to 0.5 in many bit positions. This demonstrates that the algorithm achieves a significant degree of diffusion. Although relatively lower values, such as 0.4375, are encountered in some bit positions, the overall trend is strong. Despite its simplicity, the BA algorithm produced cryptographically effective results and achieved a satisfactory SAC distribution.

The SAC performance data for the s-box generated with CSBA is shown in Table 6. An examination of the values reveals that SAC yields values close to 0.5 for a large number of bit positions. This demonstrates that the CSBA algorithm successfully achieves the capacity for small input changes to have widespread and powerful effects on the output. The overall distribution is stable, and the average performance is quite high. Because CSBA’s hybrid structure strengthens both the exploration phase and the exploitation phase, more effective results were obtained for the SAC metric.

An important statistic for evaluating the level of independence among an s-box’s output bits is BIC. The independence of output bits is essential for improving defense against linear and differential cryptanalysis assaults from the perspective of cryptographic security. Consequently, BIC nonlinearity analysis is frequently used as a crucial criterion to assess the resulting s-boxes’ cryptographic quality. Table 7 and Table 8 display the BIC nonlinearity values of the s-boxes that were produced using the CSBA and BA algorithms, respectively.

A review of Table 7 indicates that the BIC nonlinearity values obtained from the BA-generated s-box are spread in a relatively uniform manner, without significant deviations or irregularities. The values exhibit a distribution between 98 and 108.

The s-boxes generated using CSBA, which are shown in Table 8, yield values between 98 and 108 when the BIC nonlinearity results are examined. The distribution of these high values is steady.

BIC-SAC analysis jointly measures two fundamental cryptographic properties of s-boxes. BIC assesses the independence between output bits, while SAC measures the extent to which the output bits are impacted when an input bit is changed. By evaluating these two metrics together, the robustness of an s-box in terms of both propagation and independence can be understood. Table 9 and Table 10 present the BIC-SAC values for s-boxes obtained with the BA and CSBA algorithms, respectively. The results show that while both algorithms have the potential to produce secure s-boxes, CSBA provides statistically more consistent results, closer to ideal values.

Table 9 presents the BIC-SAC matrix associated with the s-box generated via the BA algorithm. The majority of the values lie between 0.48 and 0.52, a range commonly regarded as acceptable for cryptographic purposes. In summary, the observed BIC-SAC results indicate that the BA-based s-box achieves a satisfactory level of cryptographic robustness.

The BIC-SAC values obtained with the CSBA algorithm, shown in Table 10, exhibit a fairly balanced, high, and near-ideal distribution. The majority of the values fall between 0.50 and 0.52, demonstrating that the s-box is highly successful in terms of both bit-to-bit independence and the propagation effect of input-bit variation. Thanks to the hybrid structure of CSBA, the algorithm achieved high success in terms of BIC-SAC.

The Input/Output XOR distribution table is a key metric used to evaluate the resistance of an s-box to linear and differential cryptanalysis attacks. This table displays the s-box’s difference propagation behavior by showing the number of times the output differences generated in response to a given input difference are repeated. Low and balanced repeat values indicate that the s-box’s outputs are unpredictable in response to possible differences, thus providing stronger confusion (complexity) in the encryption process. Table 11 and Table 12 present the input/output XOR distributions of s-boxes generated by the BA and CSBA algorithms, respectively.

Table 11 shows the values of the XOR distribution of the s-box that was produced using the BA. Examining this table, it is observed that the repeating values are mostly 6 and 8, and less frequently 4 and 10. This distribution indicates that the differential characteristics of the s-box are largely balanced. This generally indicates a moderately balanced distribution.

Table 12 presents the XOR distribution results for the s-box produced using the CSBA method. As observed in the table, most values are concentrated around 6, 8, and 10, while lower frequencies—such as 4—appear only occasionally. The overall distribution presents a more balanced, stable, and nearly homogeneous structure compared to the BA algorithm. Examining the results confirms that the CSBA algorithm can produce s-boxes highly resistant to differential cryptanalysis. The absence of a significant gap between frequencies indicates that output differences occur with equal probability, strengthening the defense against statistical attacks.

The linear uniformity/probability of the proposed s-boxes are presented in Table 13. Linear probability is the maximum probability that an S-box can be predicted using a linear approximation, and in cryptography, s-boxes are desired to be as small as possible for their resistance to linear attacks. The produced s-boxes are extremely resistant to linear cryptanalysis attacks and offer a dependable framework for contemporary encryption algorithms, as demonstrated by the low linear probability values of 0.1328 found for both the BA and CSBA-based optimization methods in this study.

Table 14 presents a multidimensional comparison of the s-boxes generated by BA and CSBA, two different proposed chaotic-based optimization algorithms, with other heuristic optimization-based s-box designs in the literature. Six key metrics—the fundamental cryptographic security indicators of s-boxes—were compared, including SAC, nonlinearity, BIC-SAC, BIC-NL, maximum difference distribution (DDT Max), and LU. The Min, Max, and Avg values in the SAC column represent the minimum, maximum, and average SAC values between the output bits, respectively. Table 14 compares the results of s-boxes generated using only metaheuristic optimization methods. Mathematically based s-box designs are excluded due to methodological differences.

A detailed examination of Table 14 reveals that the average SAC for BA is 0.5051 and for CSBA it is 0.5000. These values show that about half of the output is impacted by single-bit changes in the input. This demonstrates that the encryption system reacts strongly to even little changes in the input and has strong diffusion features. Compared to other methods in the literature, the s-boxes obtained in this study demonstrate strong diffusion capabilities. Regarding the nonlinearity value, we obtained very high average nonlinearity values of 109.25 for BA and 109.75 for CSBA. These values are among the highest in the list compared to other studies in the literature. This illustrates how the suggested s-boxes improve cryptographic security and offer strong defense against linear cryptanalysis attacks.

Both the s-box produced by CSBA and the s-box produced by BA had BIC-SAC values of 0.5032 and 0.5040, respectively. These values indicate that the input-based independence relationships between the output bits are strongly preserved, meaning there is no predictability or pattern formation between the bits. This feature is a critical factor for secure encryption systems. In terms of BIC-NL, BA has a value of 103.14, and CSBA has a value of 103.50. This indicates that the nonlinear relationship between the output bits is sufficient and that correlation formation between blocks is prevented.

The DDT Max value obtained for the s-boxes produced in this study is 10. This value is not ideal, but it is at the same or better level than many reference values. In this sense, it is highly resistant to differential attacks. Another metric, the LP value, was found to be 0.1328125, which is similar to other literature examples. This ratio indicates a sufficient level of security against linear attacks. Consequently, this gap can be filled using the nonlinearity values of the s-boxes found in this study.

This comprehensive table demonstrates that the proposed methods are competitive with their counterparts in the literature not only in terms of individual metrics but also in terms of multiple security requirements. The generated s-boxes generally exhibit high nonlinearity, good SAC and BIC performance, low DDT, and reasonable LP values, demonstrating a strong cryptographic structure. Aydın et al. stated in their study that all s-boxes in the literature should have a minimum limit of 103 or higher for cryptographic use. An examination of Table 14 demonstrates support for this structure. This study stands out for its consistency, stability, and ability to verify the reality in the literature and compete with it [13].

Table 15 shows a comparison of the results obtained from BA and CSBA with those obtained from PSO, DE, and GA. To ensure a fair and reliable comparison, the population size for all algorithms was 30, the number of iterations was 100, and the nonlinearity value was used as the fitness value.

A detailed examination of Table 15 reveals that the CSBA method achieves better results compared to the other algorithms. For a cryptographically strong s-box, the SAC value is expected to be close to 0.5. Examining this table reveals that the result closest to this value is obtained from CSBA. Comparing the nonlinearity values reveals that the highest value is obtained first from CSBA, followed by BA. The ideal value for the BIC-SAC value, which represents the strength of the independence between the output bits, is 0.5. An examination of the table reveals that the proposed algorithms provide good results compared to the other algorithms. For the DDT Max criterion, Table 15 reveals that the worst result is obtained from DE, while all other methods yield a value of 10. Similarly, for the LU value, all five methods yielded a value of 0.138125.

## 6. Conclusions

In this study, a new hybrid method combining the global search capability of CSA and the local optimization power of BA is proposed to generate s-box structures with strong cryptographic properties. The initial population is generated using a four-dimensional Memristive Lu chaotic map to increase diversity and ensure a more balanced distribution in the solution space. The proposed chaotic hybrid CSBA effectively balances global and local search strategies, aiming to overcome the limitations encountered in classical heuristic methods.

Experimental analyses revealed that the proposed method stands out with its high nonlinearity, ideal SAC values, and strong BIC performance. The resulting s-boxes also exhibit low differential uniformity and linear prediction probability, meeting the fundamental security criteria required for symmetric encryption algorithms. In particular, it was observed that the CSBA algorithm achieves a higher average nonlinearity value and exhibits a more stable convergence behavior compared to the standard BA algorithm. Comparisons with other studies in the literature confirm that the proposed method delivers competitive and reliable results.

The integration of chaotic maps with hybrid heuristic algorithms not only produces higher-quality s-boxes but also demonstrates the applicability of this approach to practical encryption applications. Future work could include adapting the algorithm to generate larger s-boxes, testing different Memristive chaotic systems, or integrating it into real-world applications such as image encryption and IoT data protection.

## Figures and Tables

**Table 1:** The pseudocode of the initial step.

**Initialization of the Population** Set initial values a, b, c, d, x=0.1, y=0.2, z=0.3, w=0.4 **For** *i* = 1 to Populationsize (30) **do** Initialize empty list UsedValues j=1 **While** j≤S−box size (256 for 8−bit) **do** Calculate $z_{m+1}$ chaotic number $value=round\left(255\cdot z_{m+1}\right)$ **If** value not in UsedValues **then** $X_{i,j}$ = value Add value to UsedValues j=j+1 **End if** m=m+1 **End while** **End for**

**Table 2:** S-box generated by BA.

#	0	1	2	3	4	5	6	7	8	9	A	B	C	D	E	F
0	195	206	50	84	1	107	57	47	121	173	191	73	80	190	231	71
1	72	147	255	207	15	49	203	199	209	220	29	161	250	143	189	90
2	185	251	252	75	30	53	238	113	108	232	43	217	186	213	74	100
3	58	242	166	3	157	130	13	230	93	142	25	9	124	192	11	20
4	40	89	36	38	63	152	115	92	55	169	218	246	145	160	52	164
5	171	234	226	223	83	82	153	141	54	67	120	22	94	241	69	149
6	139	77	127	10	126	188	59	163	24	31	162	17	110	176	18	216
7	225	66	86	37	212	221	156	219	178	96	61	165	236	214	76	44
8	184	155	193	116	245	114	253	181	14	79	26	229	198	168	180	154
9	87	64	0	227	33	159	224	97	62	91	117	140	204	175	34	239
A	119	104	21	249	158	122	32	27	134	23	138	95	105	244	19	65
B	46	81	51	112	174	240	68	183	103	202	135	7	6	35	151	8
C	137	99	146	167	111	170	45	39	41	148	132	131	205	128	200	56
D	60	42	208	201	172	247	194	4	197	28	215	187	16	48	109	237
E	106	235	233	129	222	12	243	98	248	118	179	123	210	88	228	2
F	70	102	144	78	182	85	133	196	254	125	150	101	211	136	5	177

**Table 3:** S-box generated by CSBA.

#	0	1	2	3	4	5	6	7	8	9	A	B	C	D	E	F
0	160	220	217	32	118	99	104	203	158	135	28	125	244	2	47	76
1	59	236	149	233	138	15	140	164	16	170	193	204	176	174	94	5
2	211	115	20	75	26	120	247	25	151	65	156	215	31	152	139	214
3	98	83	7	4	251	167	166	97	58	38	90	29	66	56	50	88
4	93	180	127	128	100	110	188	0	205	92	44	69	225	191	237	71
5	112	254	206	39	64	23	162	49	178	227	108	37	173	35	234	107
6	22	153	252	245	57	43	132	1	34	129	216	102	19	224	226	119
7	248	229	30	12	218	192	182	199	74	168	163	187	79	6	175	61
8	103	159	172	146	131	207	147	196	136	212	86	219	239	73	246	190
9	121	14	111	179	80	157	177	183	84	137	197	36	209	81	105	184
A	85	72	144	77	67	18	96	124	169	208	238	109	232	3	165	240
B	52	11	63	143	62	55	250	185	255	249	145	70	21	126	141	82
C	114	148	54	171	87	40	202	221	9	235	231	106	228	123	89	189
D	230	154	68	17	122	91	161	200	95	78	243	53	194	222	210	241
E	27	41	101	117	195	155	48	33	113	13	24	42	213	253	223	116
F	201	150	46	181	134	60	51	130	186	8	45	198	142	133	10	242

**Table 4:** The proposed s-boxes’ nonlinearity scores.

*s-box*	N_1_	N_2_	N_3_	N_4_	N_5_	N_6_	N_7_	N_8_	*Min*	*Avg.*
***BA***	110	108	108	110	108	110	110	110	108	109.25
***CSBA***	110	110	110	110	110	108	110	110	108	109.75

**Table 5:** SAC of the BA s-box.

0.5313	0.5313	0.5156	0.4531	0.4688	0.4844	0.4844	0.5156
0.5000	0.4531	0.5781	0.4531	0.4688	0.4844	0.5938	0.4688
0.5313	0.5313	0.4688	0.4531	0.5156	0.5625	0.5156	0.5313
0.5313	0.5469	0.4844	0.5000	0.5938	0.5000	0.4844	0.5313
0.5469	0.4688	0.4375	0.5000	0.4375	0.5000	0.5313	0.5000
0.4844	0.5469	0.5000	0.5000	0.4844	0.4688	0.4844	0.5156
0.5156	0.5469	0.4375	0.4531	0.5625	0.5469	0.5469	0.4844
0.4844	0.5313	0.5000	0.5156	0.5156	0.5313	0.4688	0.5156

**Table 6:** SAC of the CSBA s-box.

0.5781	0.5000	0.4688	0.5469	0.5625	0.4219	0.5000	0.4531
0.5156	0.5469	0.5469	0.5625	0.4219	0.5469	0.5000	0.5469
0.5000	0.3906	0.5156	0.4375	0.5000	0.4688	0.4375	0.5156
0.5625	0.4688	0.4688	0.4844	0.5156	0.4844	0.5156	0.4688
0.5625	0.4844	0.5313	0.5313	0.4531	0.5313	0.5469	0.4375
0.4688	0.5469	0.5000	0.5469	0.4844	0.4688	0.5625	0.5156
0.5625	0.4531	0.4219	0.5469	0.5313	0.4688	0.3906	0.5000
0.5000	0.5313	0.5000	0.4844	0.5313	0.4688	0.4844	0.5000

**Table 7:** BIC Nonlinearity of the BA s-box.

---	98	100	102	106	102	104	104
98	---	104	106	106	106	106	106
100	104	---	102	102	102	100	106
102	106	102	---	104	106	102	98
106	106	102	104	---	104	102	100
102	106	102	106	104	---	102	106
104	106	100	102	102	102	---	102
104	106	106	98	100	106	102	---

**Table 8:** BIC Nonlinearity of the CSBA s-box.

---	106	104	102	100	106	104	100
106	---	104	108	106	104	108	104
104	104	---	106	96	106	102	106
102	108	106	---	98	100	106	104
100	106	96	98	---	104	104	100
106	104	106	100	104	---	104	106
104	108	102	106	104	104	---	100
100	104	106	104	100	106	100	---

**Table 9:** BIC-SAC values of the BA s-box.

---	0.4941	0.4844	0.5176	0.4922	0.5098	0.4980	0.5020
0.4941	---	0.4746	0.5000	0.4844	0.4883	0.4961	0.4922
0.4844	0.4746	---	0.4863	0.4961	0.5234	0.5234	0.5000
0.5176	0.5000	0.4863	---	0.5117	0.5195	0.4980	0.5449
0.4922	0.4844	0.4961	0.5117	---	0.5273	0.5098	0.5059
0.5098	0.4883	0.5234	0.5195	0.5273	---	0.5059	0.4922
0.4980	0.4961	0.5234	0.4980	0.5098	0.5059	---	0.5117
0.5020	0.4922	0.5000	0.5449	0.5059	0.4922	0.5117	---

**Table 10:** CSBA s-box BIC-SAC values.

---	0.5156	0.5137	0.4922	0.5059	0.5332	0.4863	0.5156
0.5156	---	0.5156	0.5000	0.5195	0.5020	0.4980	0.4902
0.5137	0.5156	---	0.5000	0.5176	0.5430	0.4922	0.4902
0.4922	0.5000	0.5000	---	0.4727	0.4883	0.4844	0.5176
0.5059	0.5195	0.5176	0.4727	---	0.4961	0.5156	0.5000
0.5332	0.5020	0.5430	0.4883	0.4961	---	0.5117	0.4922
0.4863	0.4980	0.4922	0.4844	0.5156	0.5117	---	0.5039
0.5156	0.4902	0.4902	0.5176	0.5000	0.4922	0.5039	---

**Table 11:** Input/output XOR distribution table of BA.

6	8	8	8	6	6	4	6	8	6	8	8	6
8	6	6	6	10	6	4	6	6	6	6	8	6
6	8	6	6	8	6	6	6	6	8	6	6	8
8	6	6	8	6	6	6	6	6	8	8	4	8
8	8	6	8	6	8	8	6	6	8	8	6	10
6	8	8	8	6	6	8	6	6	8	6	6	8
6	8	6	8	8	6	8	6	6	6	6	6	8
6	6	6	8	6	6	8	6	6	6	6	4	6
6	8	8	8	6	6	8	6	6	8	6	6	6
6	6	6	6	6	8	6	8	8	8	6	6	8
6	6	6	6	8	6	6	8	6	8	6	6	6
6	6	6	6	8	8	8	6	6	6	6	6	6
6	6	8	6	6	6	8	6	6	6	6	8	8
6	6	8	6	6	8	8	6	6	6	6	8	6
8	6	8	6	6	4	8	6	8	8	6	6	6
8	6	6	8	8	8	4	8	8	8	6	8	6

**Table 12:** Input/output XOR distribution table of CSBA.

8	8	8	6	8	6	6	6	6	6	6	8	6
6	6	6	6	8	10	6	6	6	6	6	6	6
6	6	8	6	6	6	6	8	8	8	8	6	8
6	6	8	6	8	6	10	6	8	6	8	4	8
6	8	8	8	8	6	6	6	8	6	8	8	6
6	6	6	6	8	6	6	6	6	8	6	6	8
8	8	6	6	8	4	8	6	6	6	6	4	6
6	8	8	6	8	6	6	10	6	8	6	6	6
6	6	8	6	4	6	6	6	6	6	6	6	6
6	8	6	6	6	8	6	6	6	6	6	8	6
6	6	6	6	8	6	8	8	6	6	8	6	6
8	6	6	6	6	8	8	6	6	6	8	10	6
6	8	6	6	6	8	6	6	8	4	6	6	6
8	10	8	6	8	6	8	6	6	8	6	6	6
10	6	6	8	6	4	6	6	6	6	6	8	6
6	8	8	8	6	6	6	6	6	8	6	8	8

**Table 13:** Linear uniformity/probability of proposed s-boxes.

s-box	Linear Uniformity/Probability
**BA**	162/0.1328125
**CSBA**	162/0.1328125

**Table 14:** Comparison of optimization techniques using s-boxes based on chaos.

	SAC			Nonlinearity			BIC-SAC	BIC-NL	DDT Max	LU
Methods	Min	Max	Avg	Min	Max	Avg				
[10]	0.4063	0.5781	0.4995	106	110	108.50	0.5016	103.86	10	0.1328125
[17]	0.4063	0.5781	0.5025	102	108	105.50	0.5064	105.36	10	0.1171875
[18]	0.4531	0.5625	0.5049	112	112	112.00	0.5046	112.00	4	0.0625000
[20]	0.4219	0.6094	0.5029	106	108	106.50	0.5012	106.14	8	0.0937500
[21]	0.4219	0.6406	0.5127	104	106	105.00	0.4997	106.14	10	0.0937500
[22]	0.3906	0.6406	0.5027	90	104	99.75	0.5057	100.50	64	0.1406250
[23]	0.3906	0.5938	0.5007	104	110	107.00	0.5020	104.50	10	0.1250000
[24]	0.4063	0.6094	0.4981	106	108	107.00	0.5029	103.14	12	0.1250000
[25]	0.4375	0.5938	0.5081	100	108	105.00	0.5035	103.36	10	0.1250000
[26]	0.4219	0.6094	0.5003	104	108	105.75	0.4992	106.21	8	0.0937500
[27]	0.4219	0.5781	0.5022	110	112	111.25	0.5036	103.79	10	0.1328125
[28]	0.4063	0.5781	0.4995	110	112	110.50	0.5024	103.14	10	0.1484375
[29]	0.3750	0.5938	0.4937	108	110	109.00	0.5057	103.86	10	0.1250000
[30]	0.3750	0.5625	0.5017	106	110	109.25	0.4991	104.07	10	0.1250000
[31]	0.4063	0.6406	0.5037	104	108	106.5	0.4995	102.86	10	0.140625
BA	0.4375	0.5938	0.5051	108	110	109.25	0.5032	103.14	10	0.1328125
CSBA	0.3906	0.5781	0.5000	108	110	109.75	0.5040	103.50	10	0.1328125

**Table 15:** Comparison of the proposed BA and CSBA with GA, PSO, and DE under identical test conditions.

	SAC			Nonlinearity			BIC-SAC	BIC-NL	DDT Max	LU
Methods	Min	Max	Avg	Min	Max	Avg				
PSO	0.3906	0.5938	0.5044	102	108	105.5	0.4992	103.57	10	0.1328125
DE	0.4219	0.5938	0.5049	104	108	106.5	0.504	103.93	12	0.1328125
GA	0.4219	0.625	0.501	104	108	106.75	0.5055	103.57	10	0.1328125
BA	0.4375	0.5938	0.5051	108	110	109.25	0.5032	103.14	10	0.1328125
CSBA	0.3906	0.5781	0.5000	108	110	109.75	0.5040	103.50	10	0.1328125

## Data Availability

No external data were used in this paper, and all the data generated are part of the paper.
